# Supplementary figures and images for: Structural features stabilized by divalent cation coordination within hepatitis E virus ORF1 are critical for viral replication
Source: eLife. 2023 Feb 28;12:e80529. doi: 10.7554/eLife.80529 (PMC9977285; doi:10.7554/eLife.80529)

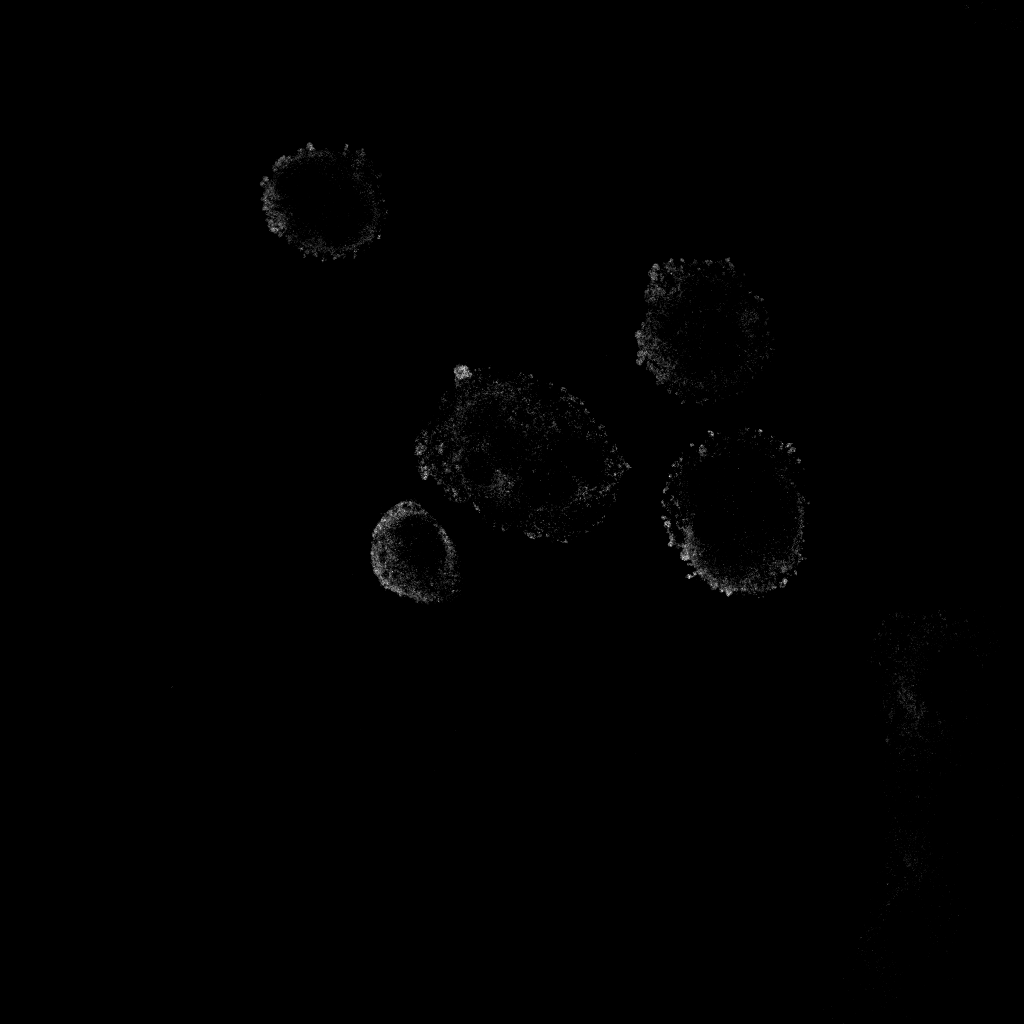

Supplement: Source data 1. — These files are the best ranked (ranked 0) predictions generated by AlphaFold of HEV ORF1, its associated point mutants, and the hepatitis A virus (HAV) 3 C protease. [file elife-80529-data1.zip › Figure 7 Source Data/HA_C3-WT_NoTag_F_20220405.tif]

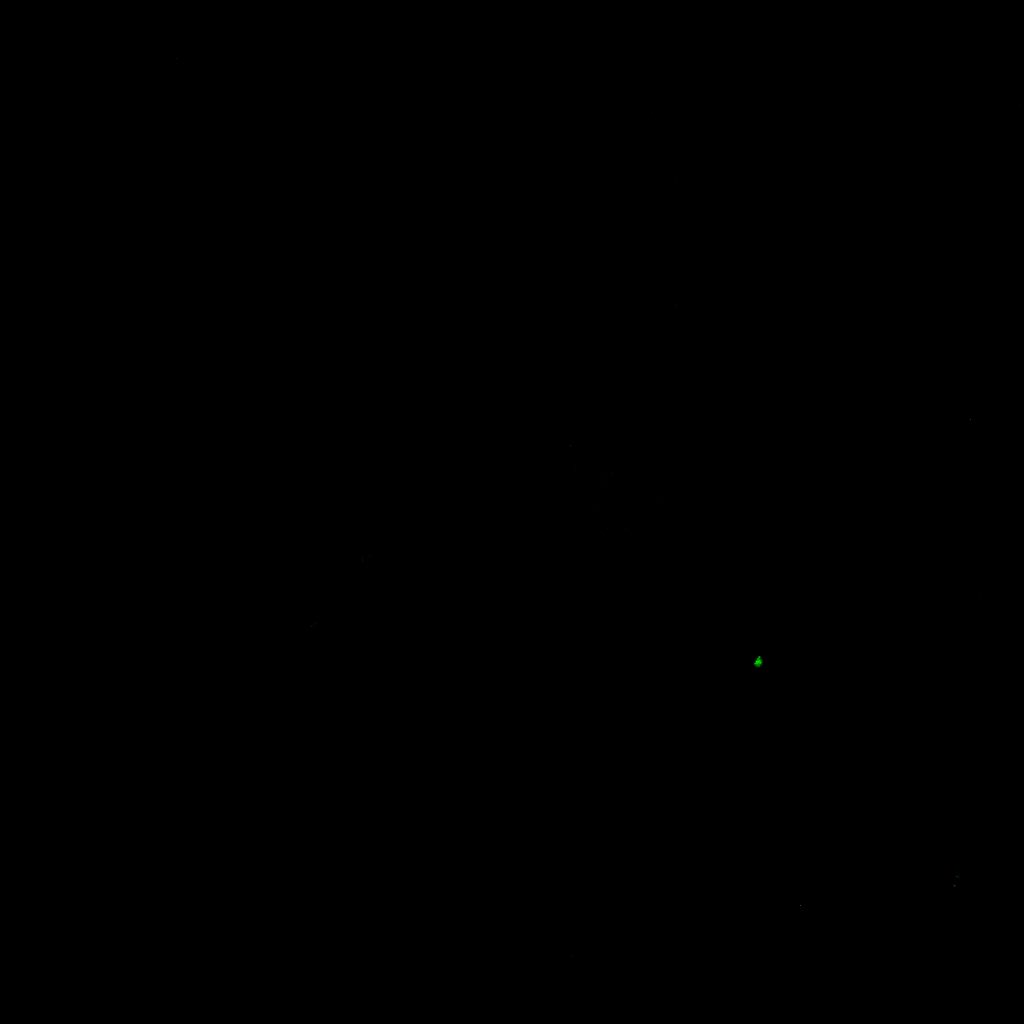

Supplement: Source data 1. — These files are the best ranked (ranked 0) predictions generated by AlphaFold of HEV ORF1, its associated point mutants, and the hepatitis A virus (HAV) 3 C protease. [file elife-80529-data1.zip › Figure 7 Source Data/zsGreen_C2-Mock_duplicate1_40x_C_20220405.tif]

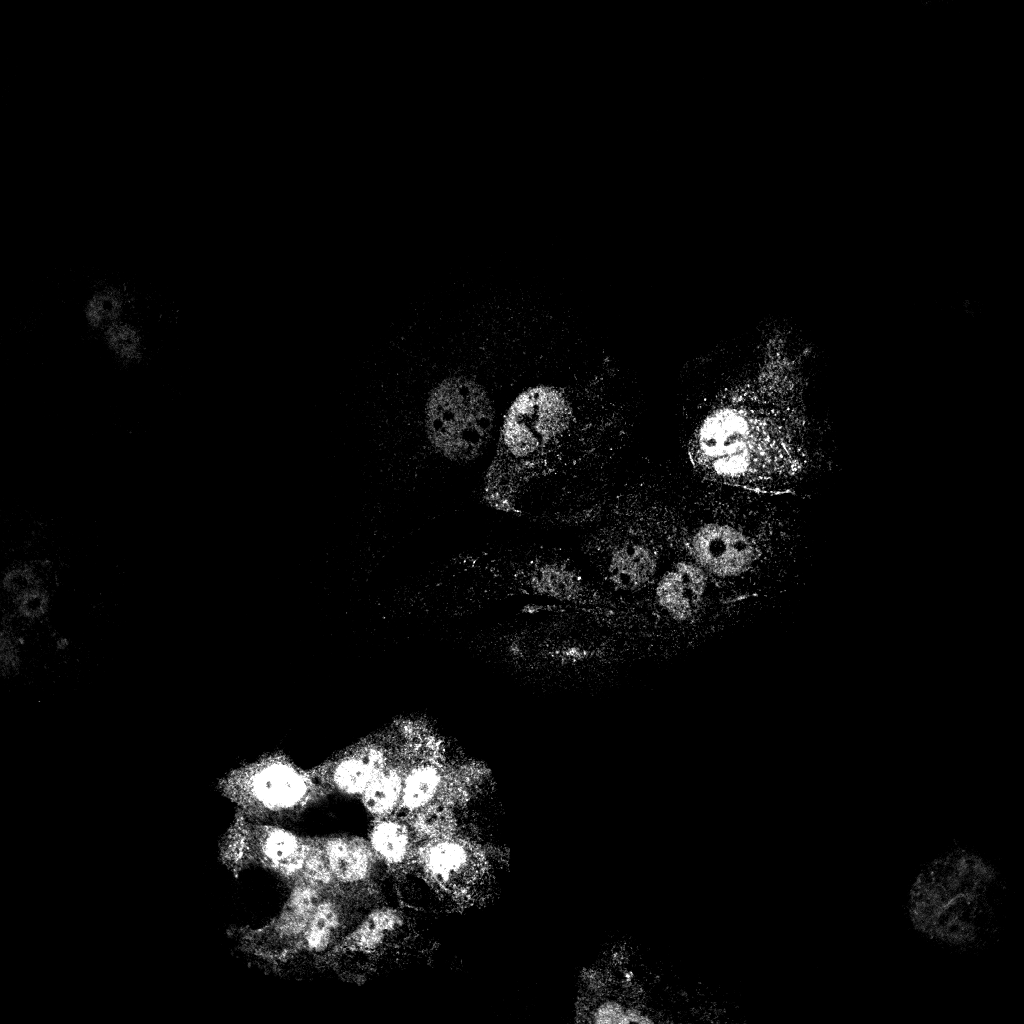

Supplement: Source data 1. — These files are the best ranked (ranked 0) predictions generated by AlphaFold of HEV ORF1, its associated point mutants, and the hepatitis A virus (HAV) 3 C protease. [file elife-80529-data1.zip › Figure 7 Source Data/HA_C3-L-Ha-L_C563A_C_20220406.tif]

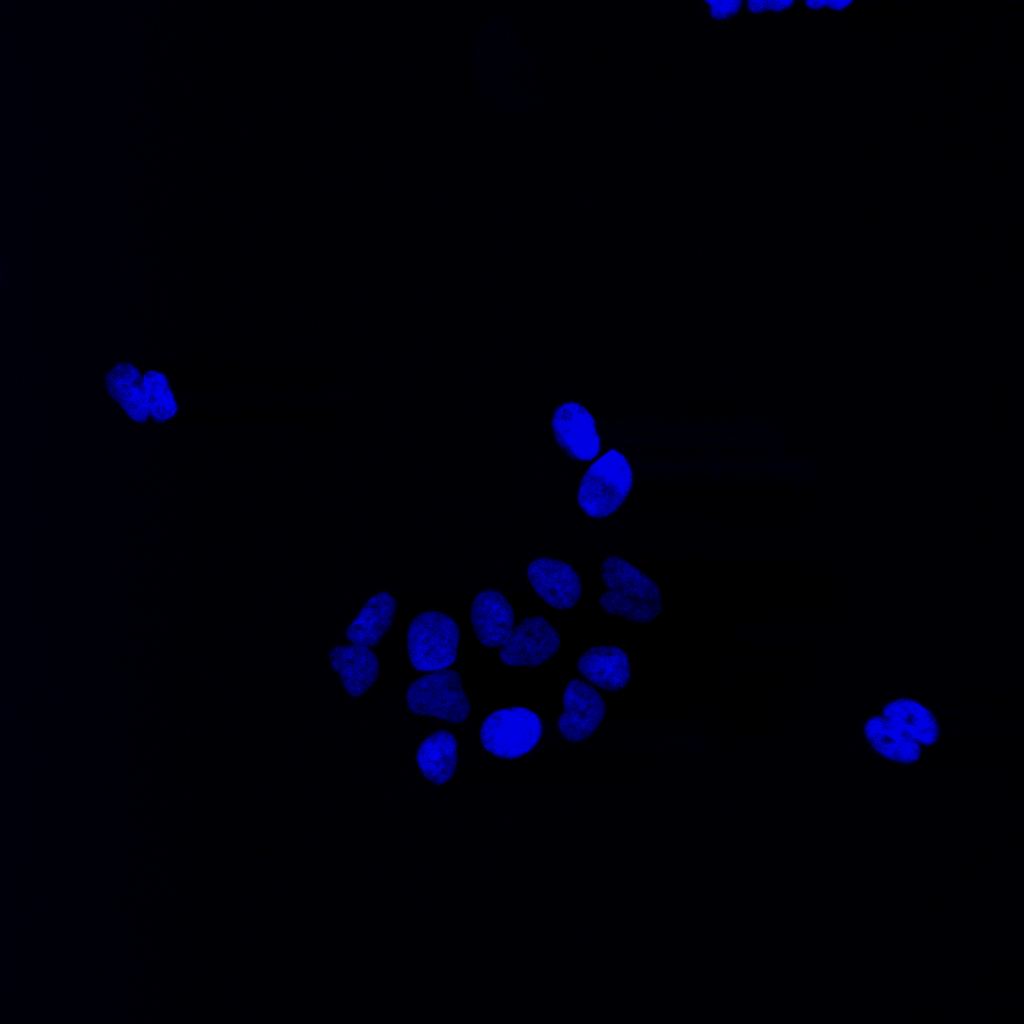

Supplement: Source data 1. — These files are the best ranked (ranked 0) predictions generated by AlphaFold of HEV ORF1, its associated point mutants, and the hepatitis A virus (HAV) 3 C protease. [file elife-80529-data1.zip › Figure 7 Source Data/Hoechst_C1-L-Ha-L_WT_E_20220405.tif]

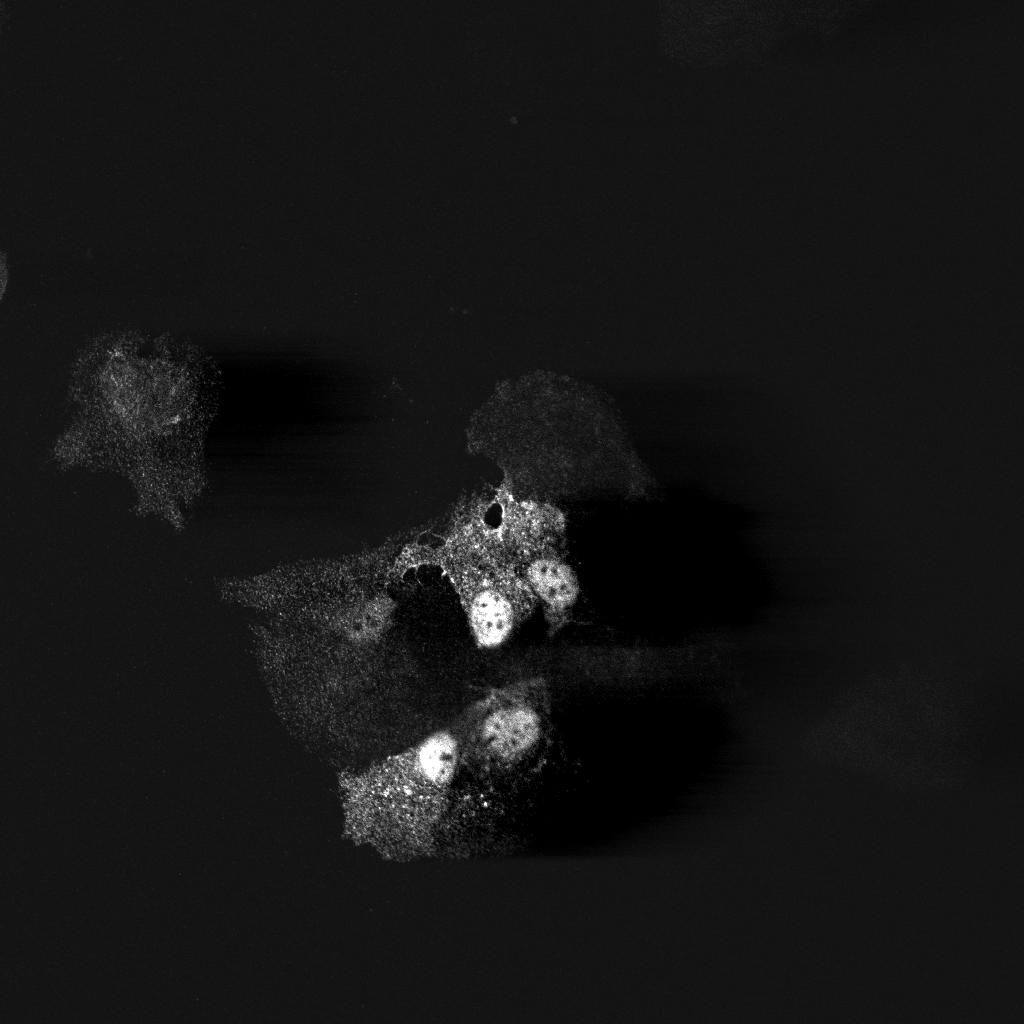

Supplement: Source data 1. — These files are the best ranked (ranked 0) predictions generated by AlphaFold of HEV ORF1, its associated point mutants, and the hepatitis A virus (HAV) 3 C protease. [file elife-80529-data1.zip › Figure 7 Source Data/HA_C3-L-Ha-L_WT_E_20220405.tif]

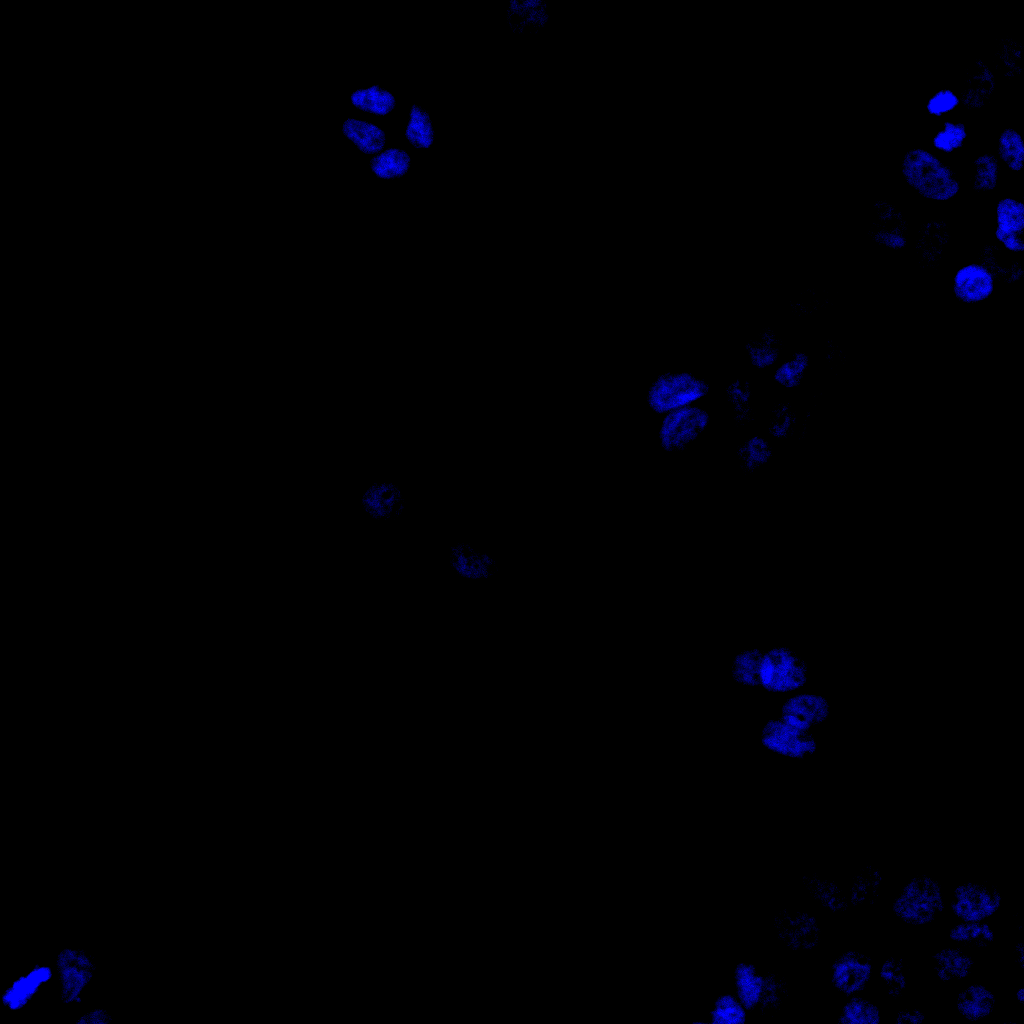

Supplement: Source data 1. — These files are the best ranked (ranked 0) predictions generated by AlphaFold of HEV ORF1, its associated point mutants, and the hepatitis A virus (HAV) 3 C protease. [file elife-80529-data1.zip › Figure 7 Source Data/Hoechst_C1-L-HA-L_H249A_C_20220405.tif]

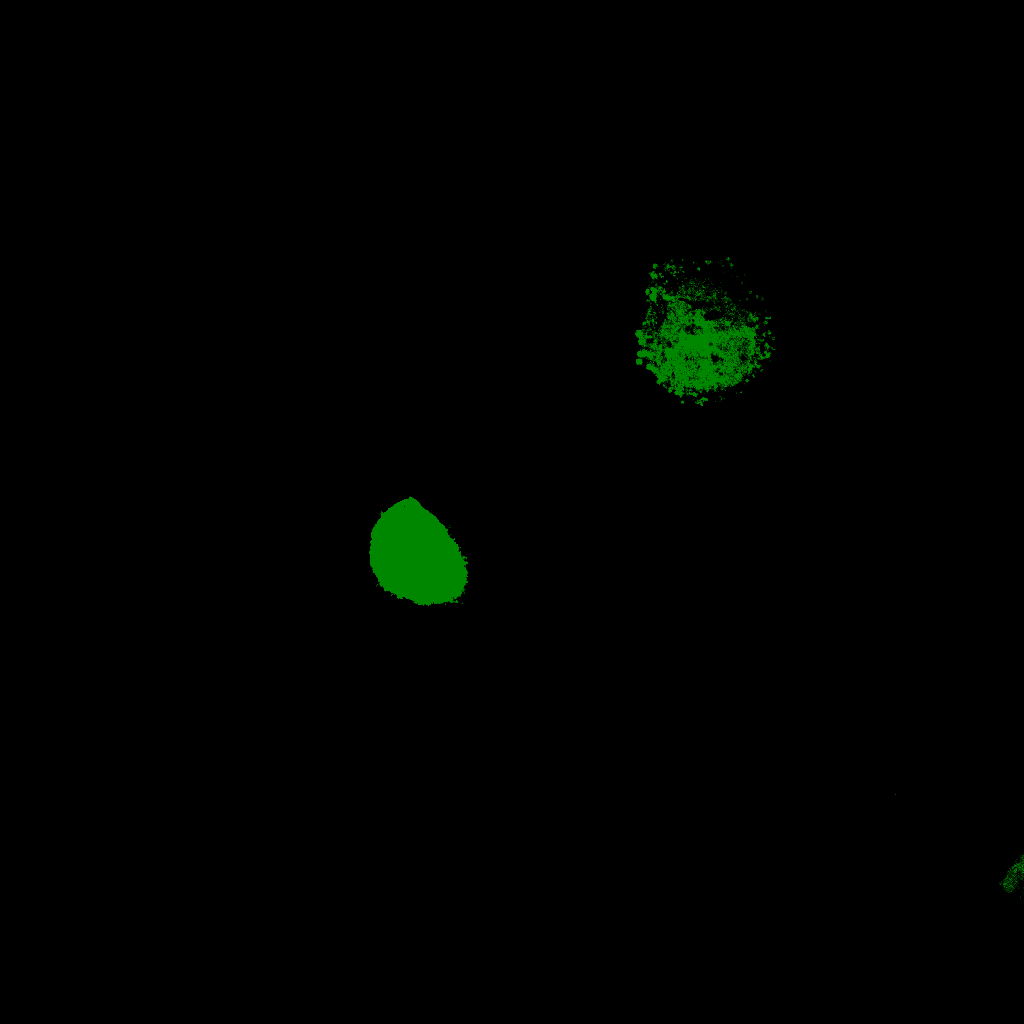

Supplement: Source data 1. — These files are the best ranked (ranked 0) predictions generated by AlphaFold of HEV ORF1, its associated point mutants, and the hepatitis A virus (HAV) 3 C protease. [file elife-80529-data1.zip › Figure 7 Source Data/zsGreen_C2-WT_NoTag_F_20220405.tif]

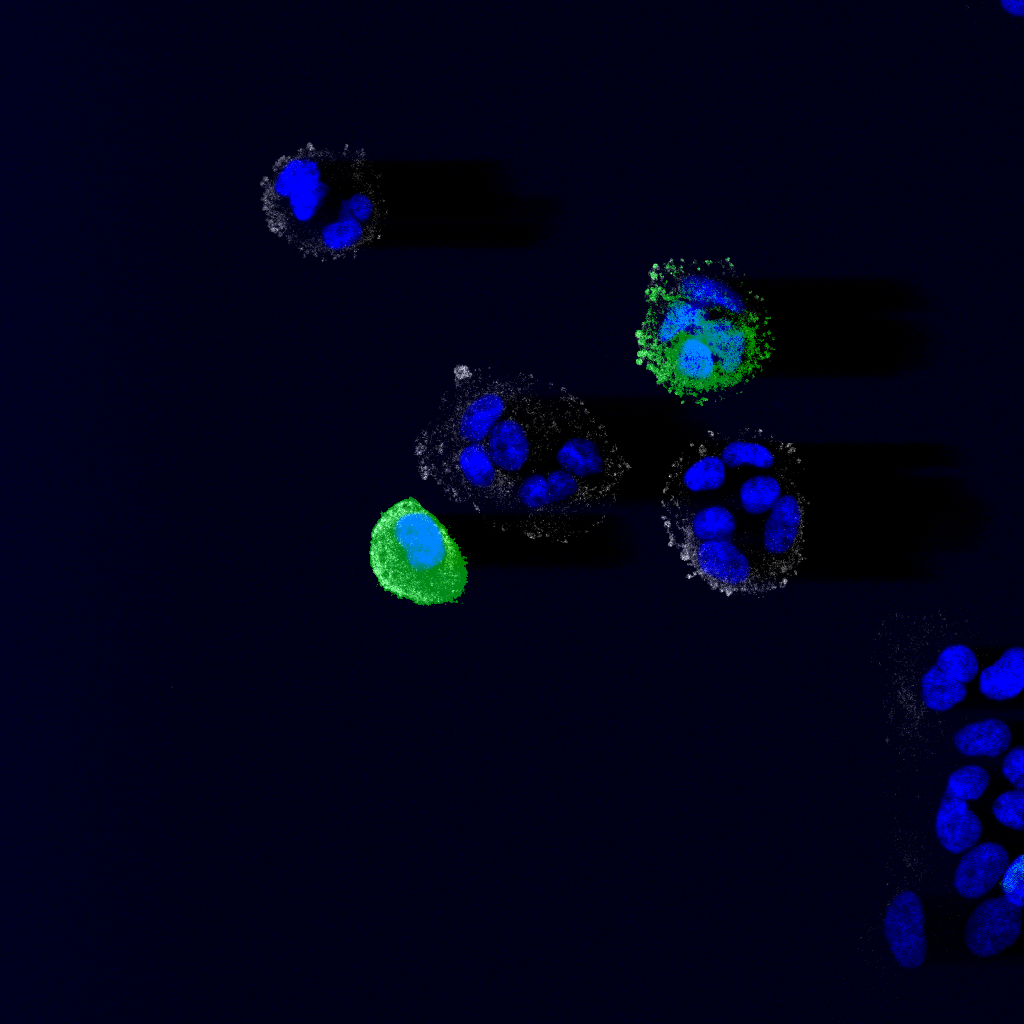

Supplement: Source data 1. — These files are the best ranked (ranked 0) predictions generated by AlphaFold of HEV ORF1, its associated point mutants, and the hepatitis A virus (HAV) 3 C protease. [file elife-80529-data1.zip › Figure 7 Source Data/Merge_WT_NoTag_F_20220405.nd2 (RGB).tif]

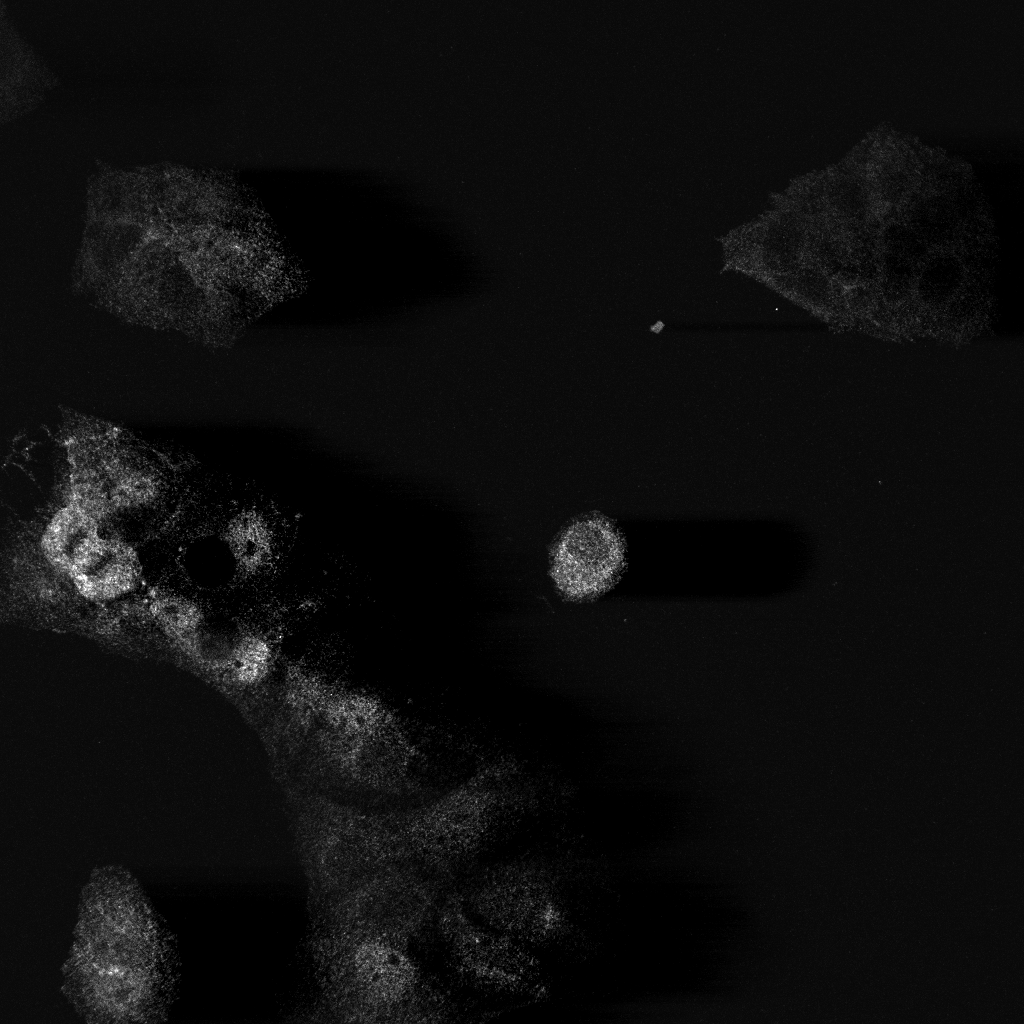

Supplement: Source data 1. — These files are the best ranked (ranked 0) predictions generated by AlphaFold of HEV ORF1, its associated point mutants, and the hepatitis A virus (HAV) 3 C protease. [file elife-80529-data1.zip › Figure 7 Source Data/HA_C3-L-HA-L_D248A_A_20220405.tif]

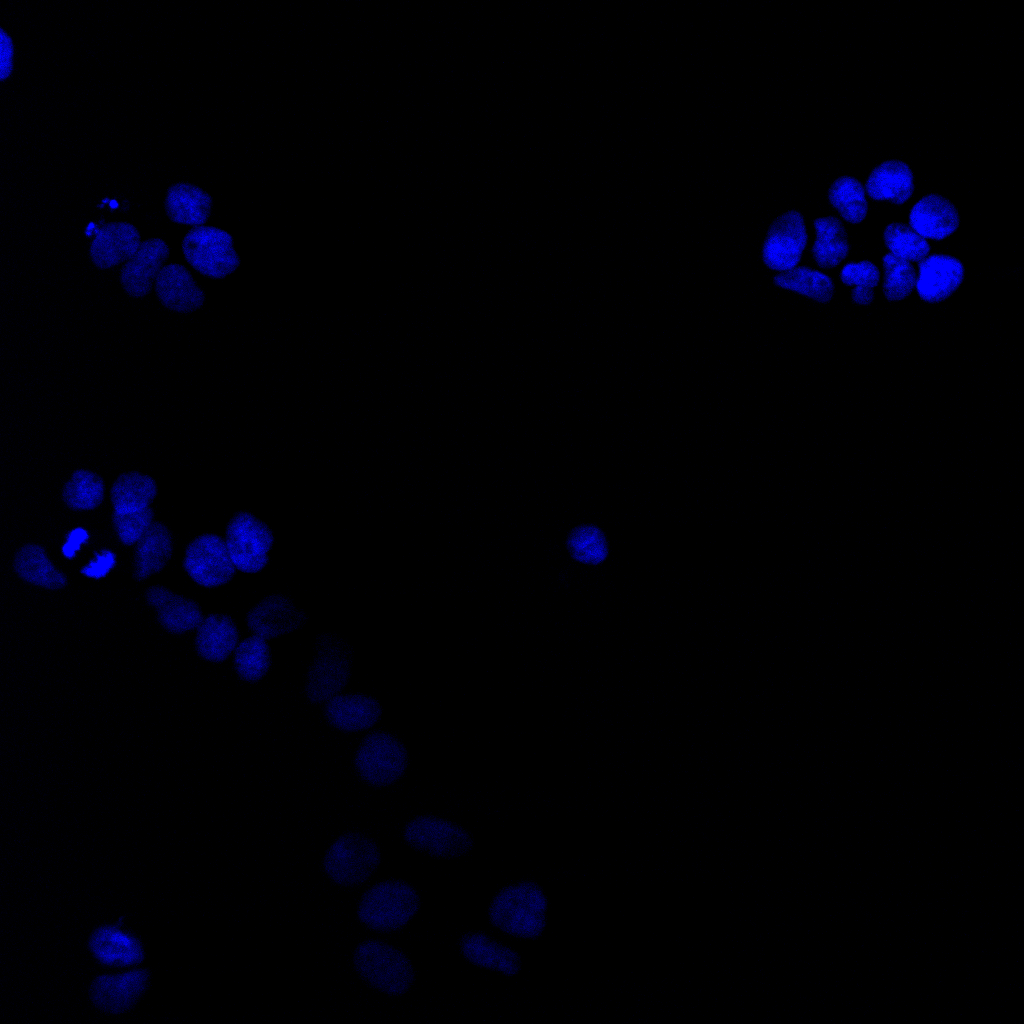

Supplement: Source data 1. — These files are the best ranked (ranked 0) predictions generated by AlphaFold of HEV ORF1, its associated point mutants, and the hepatitis A virus (HAV) 3 C protease. [file elife-80529-data1.zip › Figure 7 Source Data/Hoechst_C1-L-HA-L_D248A_A_20220405.tif]

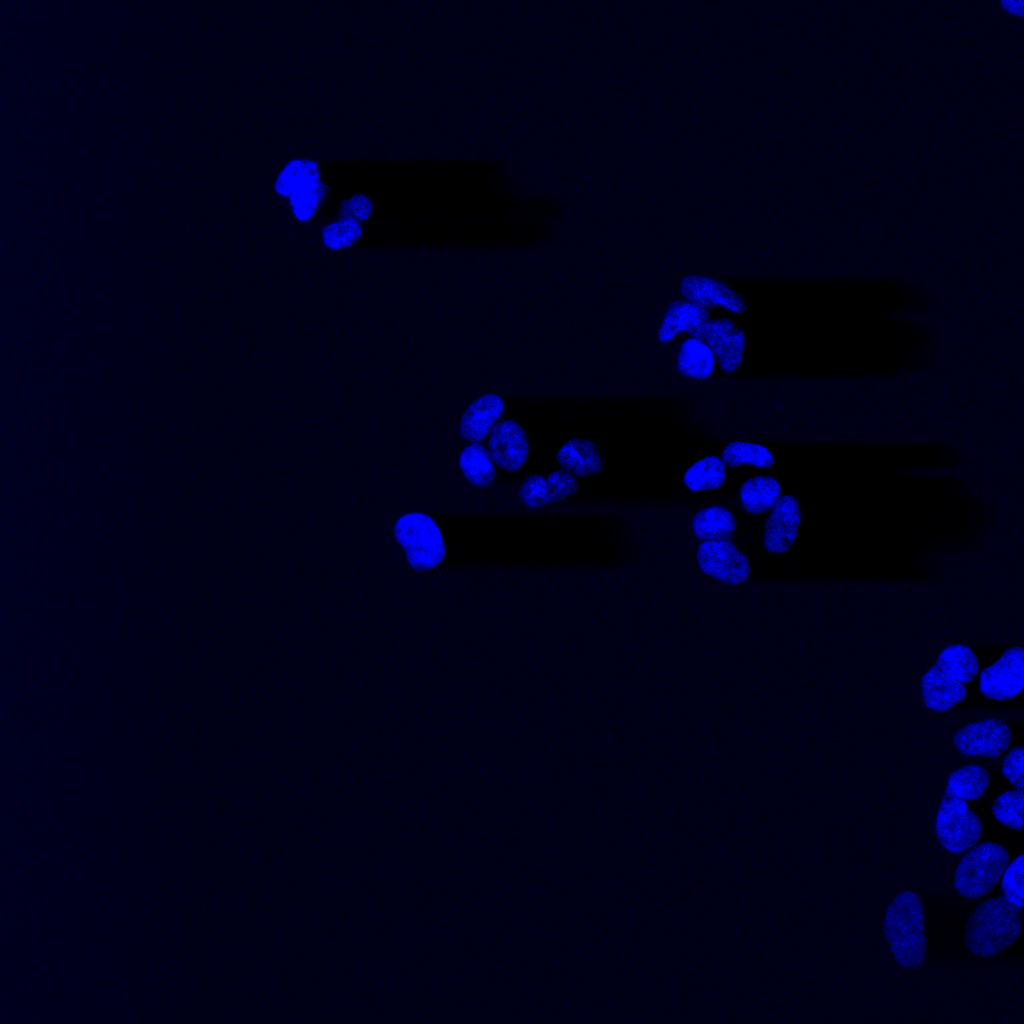

Supplement: Source data 1. — These files are the best ranked (ranked 0) predictions generated by AlphaFold of HEV ORF1, its associated point mutants, and the hepatitis A virus (HAV) 3 C protease. [file elife-80529-data1.zip › Figure 7 Source Data/Hoechst_C1-WT_NoTag_F_20220405.tif]

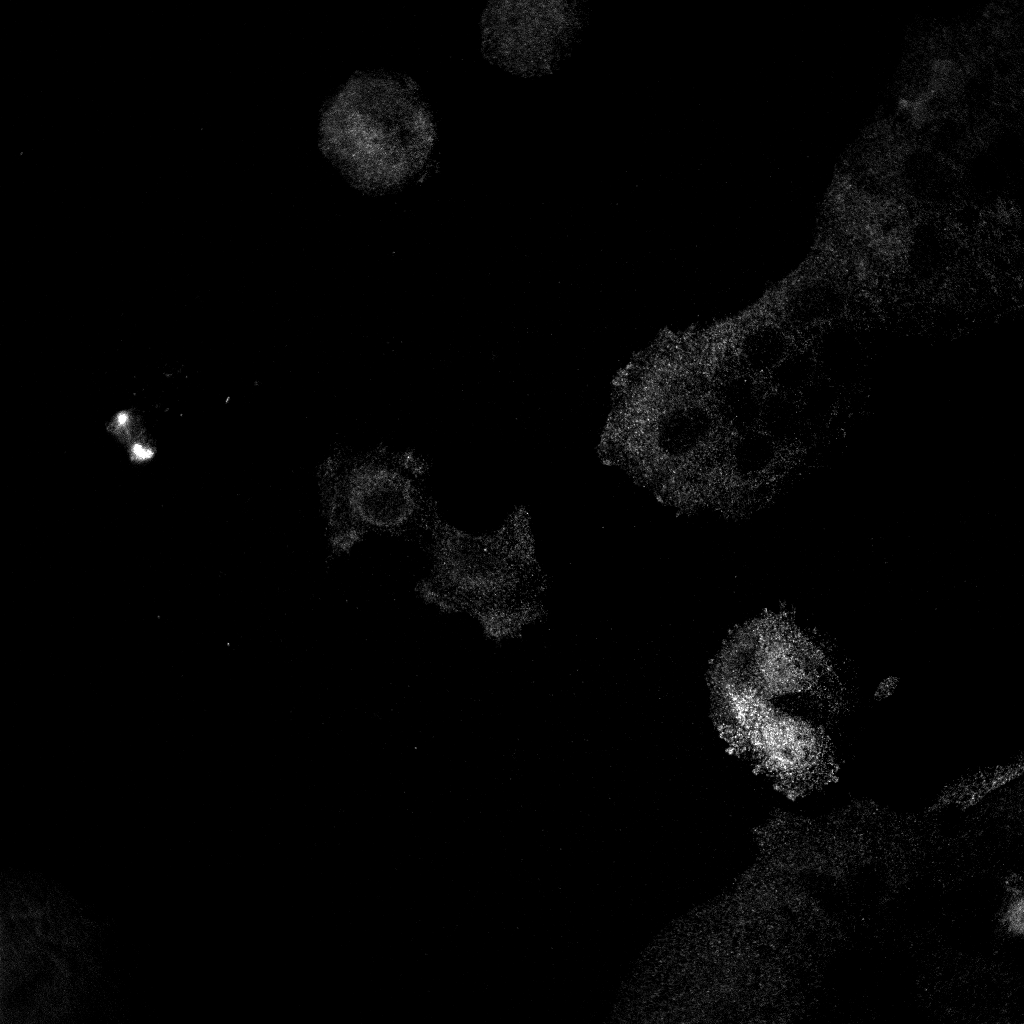

Supplement: Source data 1. — These files are the best ranked (ranked 0) predictions generated by AlphaFold of HEV ORF1, its associated point mutants, and the hepatitis A virus (HAV) 3 C protease. [file elife-80529-data1.zip › Figure 7 Source Data/HA_C3-L-HA-L_H249A_C_20220405.tif]

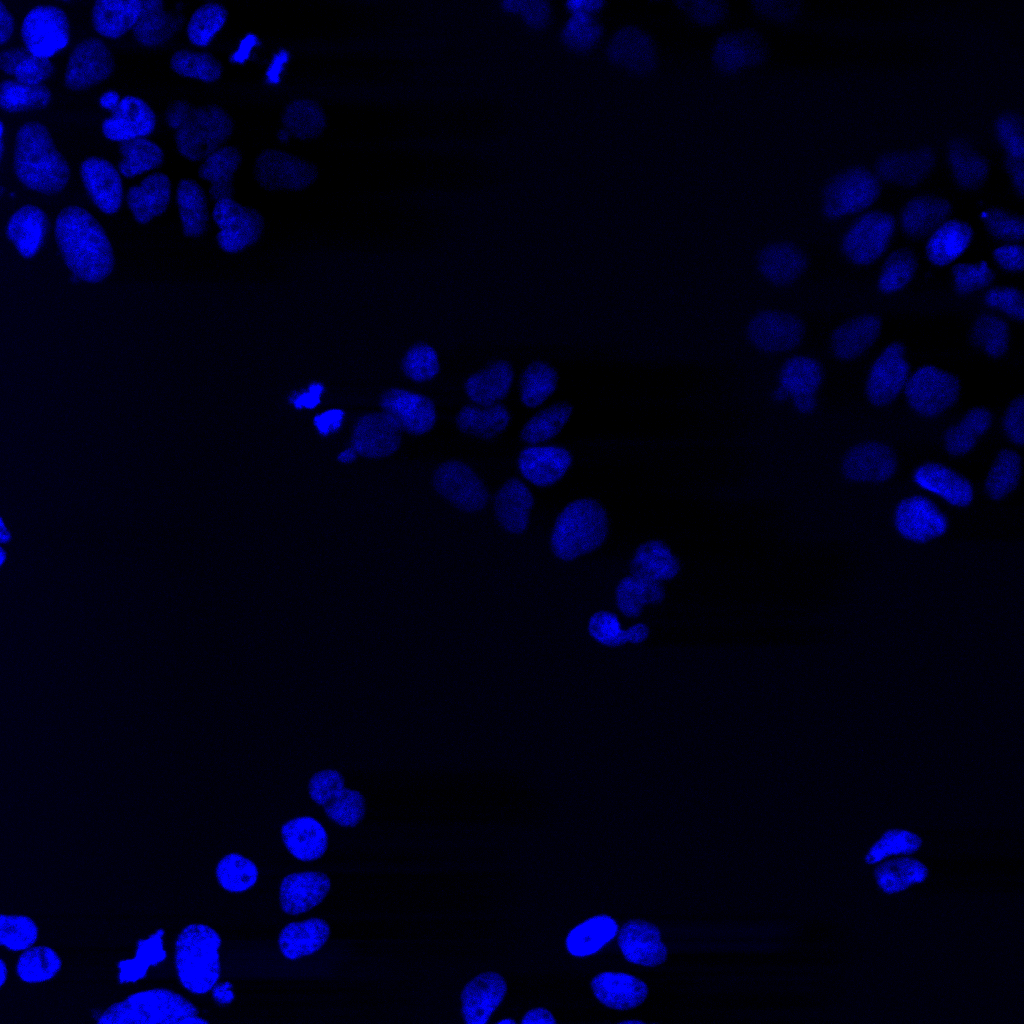

Supplement: Source data 1. — These files are the best ranked (ranked 0) predictions generated by AlphaFold of HEV ORF1, its associated point mutants, and the hepatitis A virus (HAV) 3 C protease. [file elife-80529-data1.zip › Figure 7 Source Data/Hoechst_C1-L-HA-L_C483A_40x_F_20220405.tif]

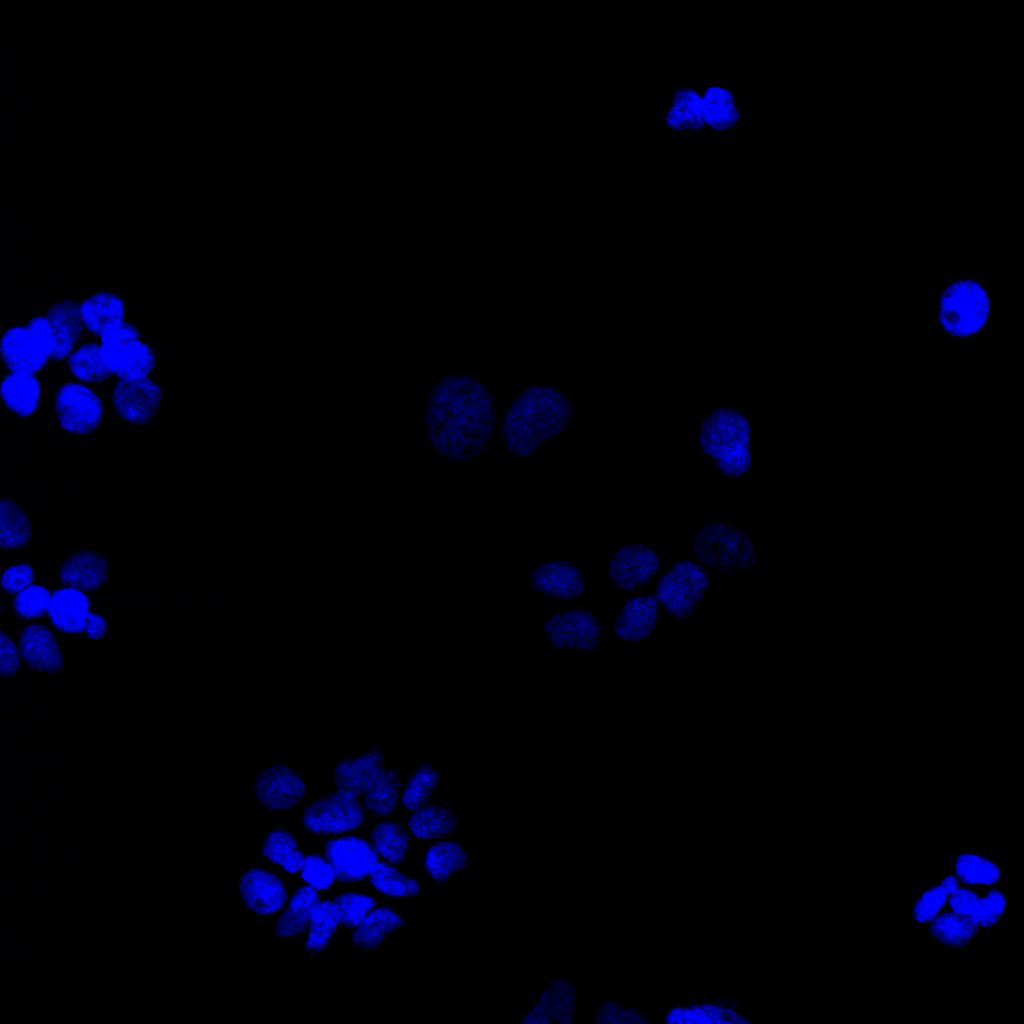

Supplement: Source data 1. — These files are the best ranked (ranked 0) predictions generated by AlphaFold of HEV ORF1, its associated point mutants, and the hepatitis A virus (HAV) 3 C protease. [file elife-80529-data1.zip › Figure 7 Source Data/Hoechst_C1-L-Ha-L_C563A_C_20220406.tif]

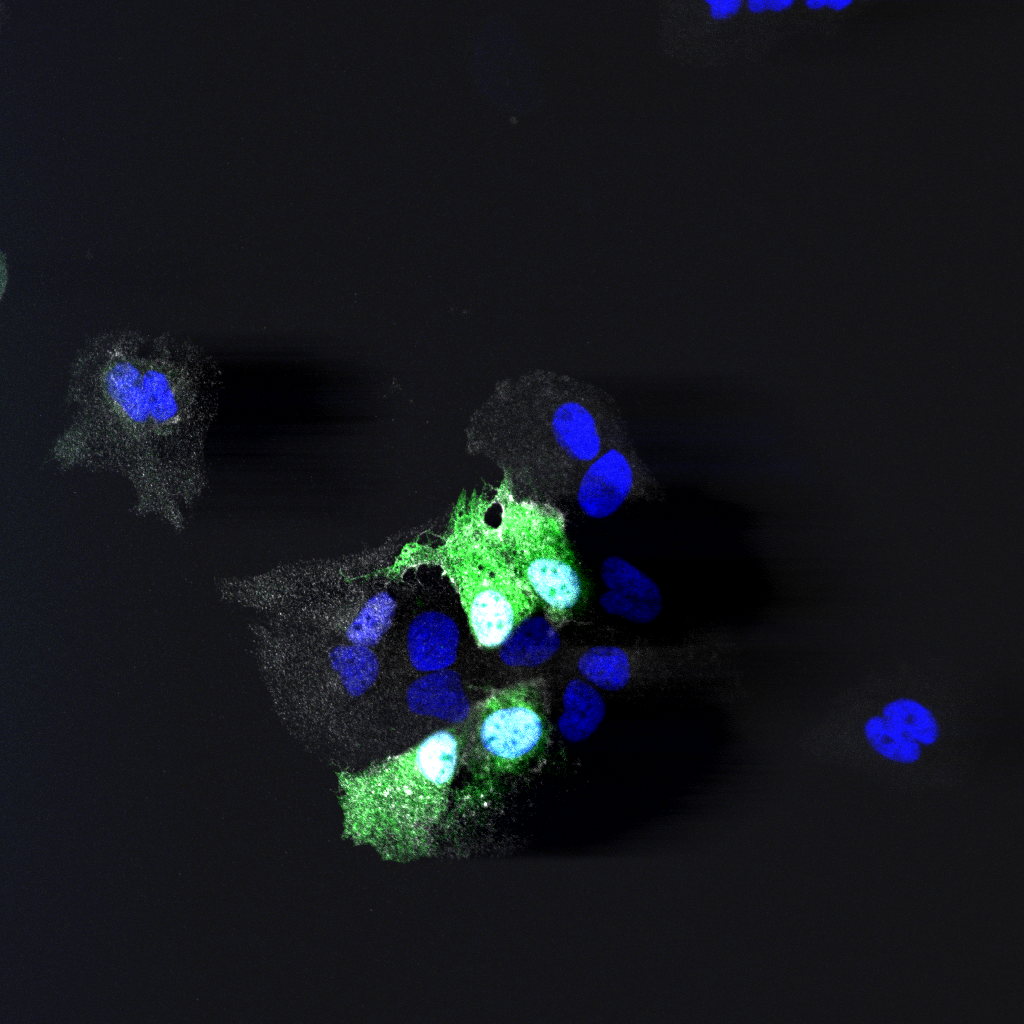

Supplement: Source data 1. — These files are the best ranked (ranked 0) predictions generated by AlphaFold of HEV ORF1, its associated point mutants, and the hepatitis A virus (HAV) 3 C protease. [file elife-80529-data1.zip › Figure 7 Source Data/Merge_L-Ha-L_WT_E_20220405.nd2 (RGB).tif]

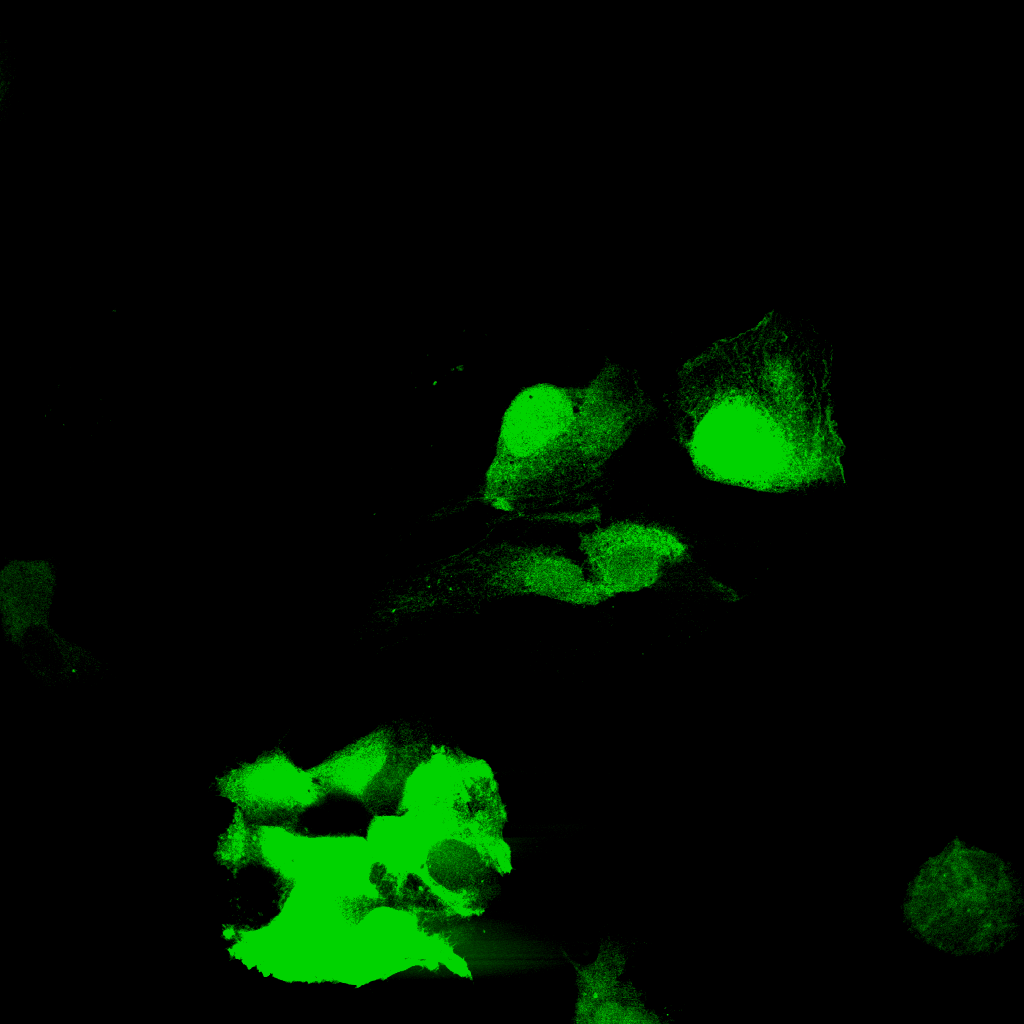

Supplement: Source data 1. — These files are the best ranked (ranked 0) predictions generated by AlphaFold of HEV ORF1, its associated point mutants, and the hepatitis A virus (HAV) 3 C protease. [file elife-80529-data1.zip › Figure 7 Source Data/zsGreen_C2-L-Ha-L_C563A_C_20220406.tif]

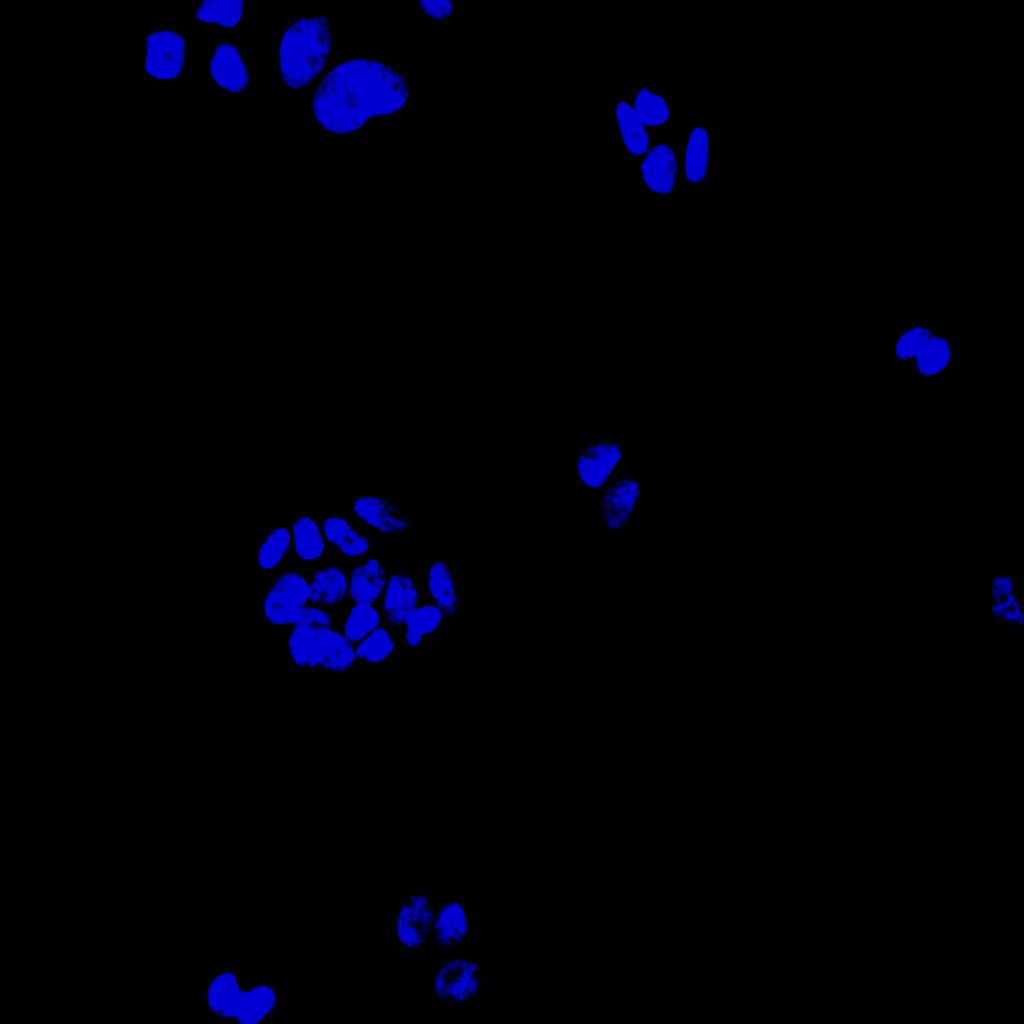

Supplement: Source data 1. — These files are the best ranked (ranked 0) predictions generated by AlphaFold of HEV ORF1, its associated point mutants, and the hepatitis A virus (HAV) 3 C protease. [file elife-80529-data1.zip › Figure 7 Source Data/Hoechst_C1-Mock_duplicate1_40x_C_20220405.tif]

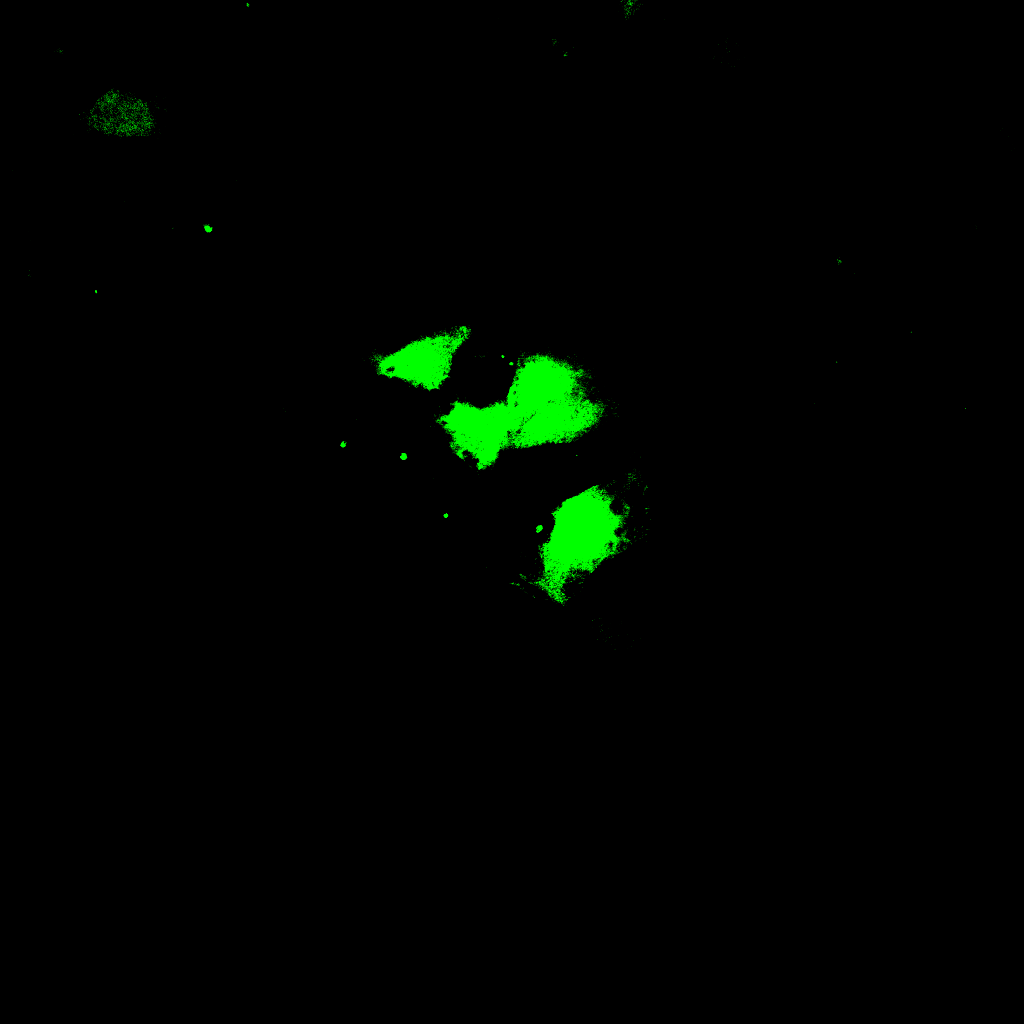

Supplement: Source data 1. — These files are the best ranked (ranked 0) predictions generated by AlphaFold of HEV ORF1, its associated point mutants, and the hepatitis A virus (HAV) 3 C protease. [file elife-80529-data1.zip › Figure 7 Source Data/zsGreen_C2-L-HA-L_C483A_40x_F_20220405.tif]

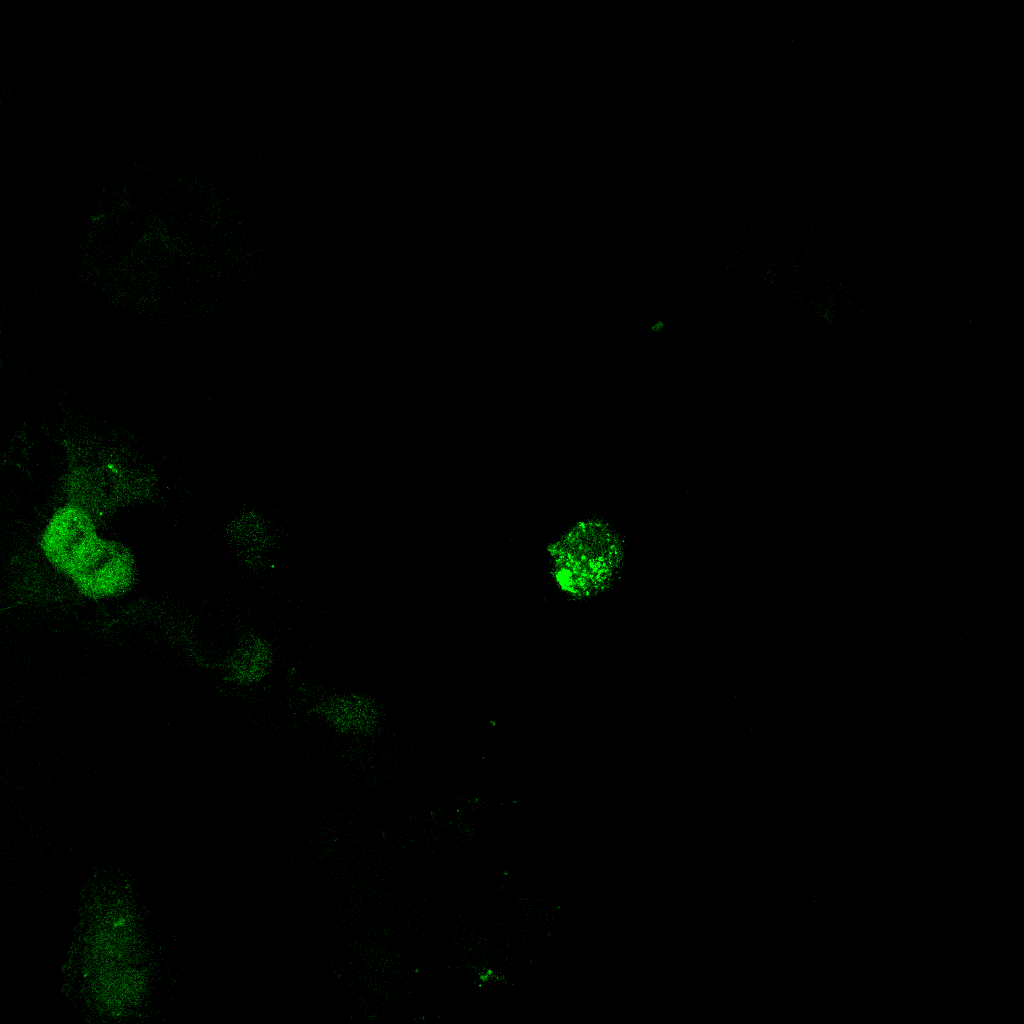

Supplement: Source data 1. — These files are the best ranked (ranked 0) predictions generated by AlphaFold of HEV ORF1, its associated point mutants, and the hepatitis A virus (HAV) 3 C protease. [file elife-80529-data1.zip › Figure 7 Source Data/zsGreen_C2-L-HA-L_D248A_A_20220405.tif]

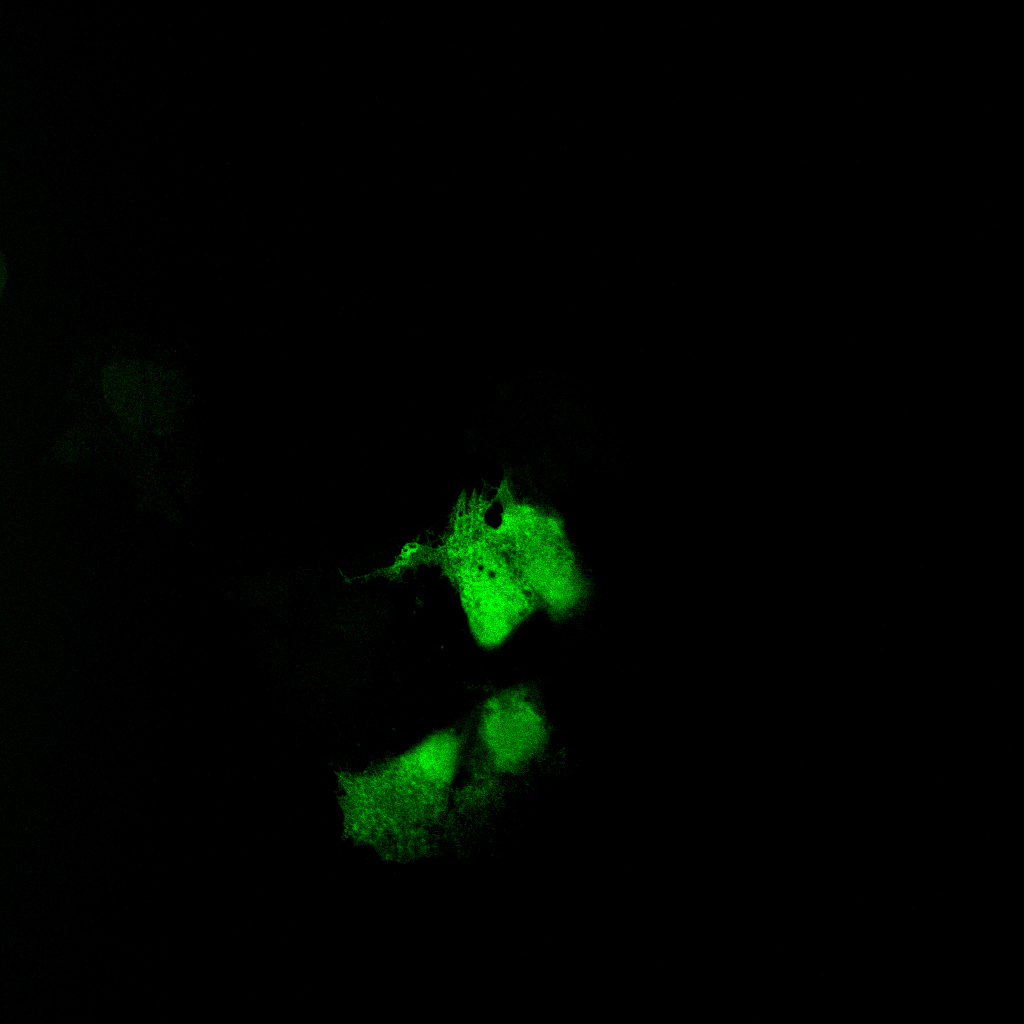

Supplement: Source data 1. — These files are the best ranked (ranked 0) predictions generated by AlphaFold of HEV ORF1, its associated point mutants, and the hepatitis A virus (HAV) 3 C protease. [file elife-80529-data1.zip › Figure 7 Source Data/zsGreen_C2-L-Ha-L_WT_E_20220405.tif]

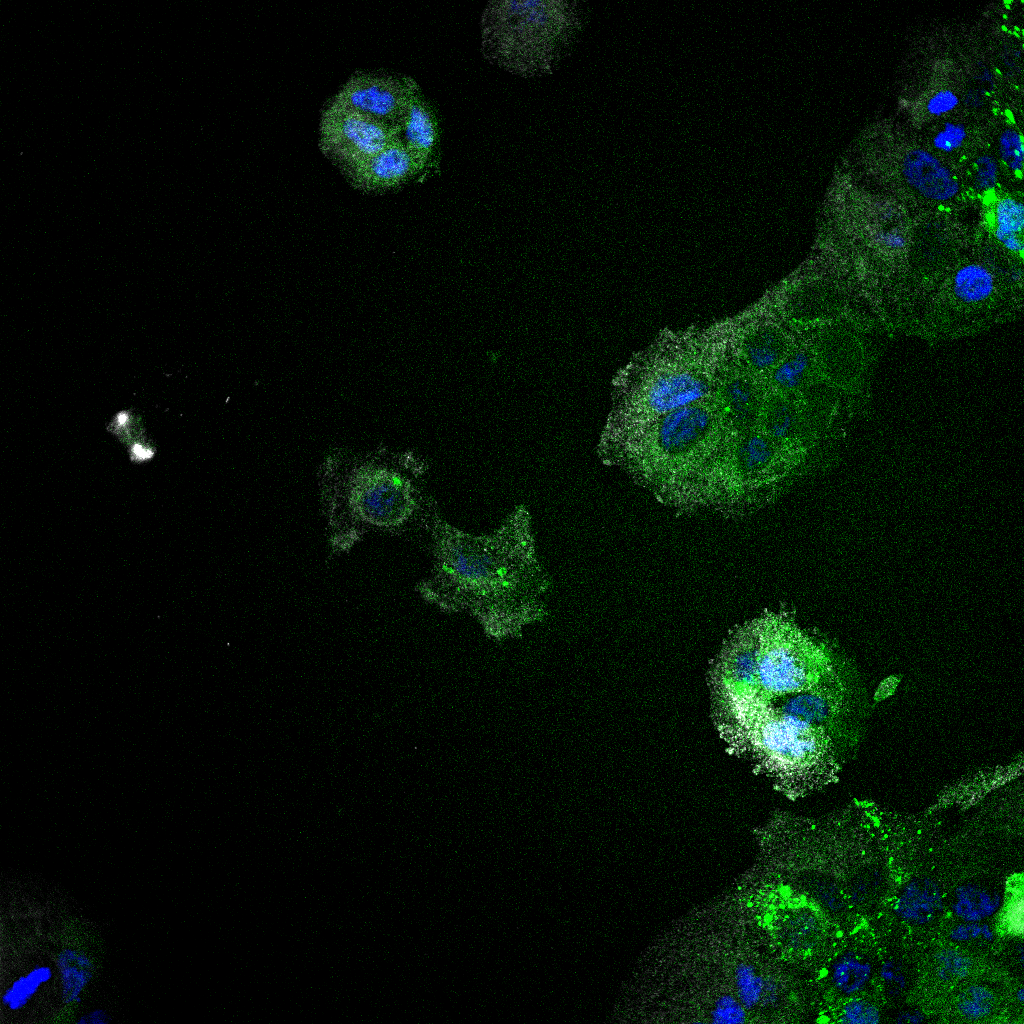

Supplement: Source data 1. — These files are the best ranked (ranked 0) predictions generated by AlphaFold of HEV ORF1, its associated point mutants, and the hepatitis A virus (HAV) 3 C protease. [file elife-80529-data1.zip › Figure 7 Source Data/Merge_L-HA-L_H249A_C_20220405.nd2 (RGB).tif]

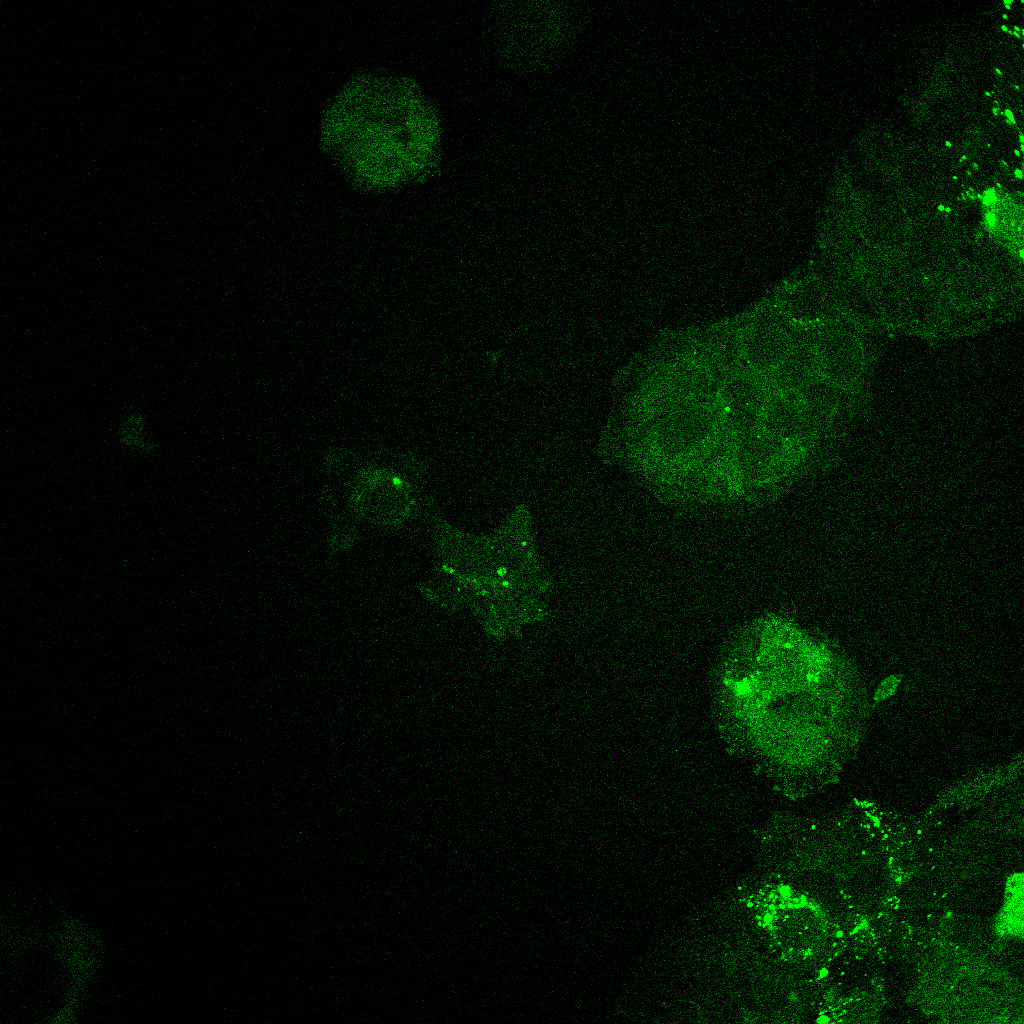

Supplement: Source data 1. — These files are the best ranked (ranked 0) predictions generated by AlphaFold of HEV ORF1, its associated point mutants, and the hepatitis A virus (HAV) 3 C protease. [file elife-80529-data1.zip › Figure 7 Source Data/zsGreen_C2-L-HA-L_H249A_C_20220405.tif]

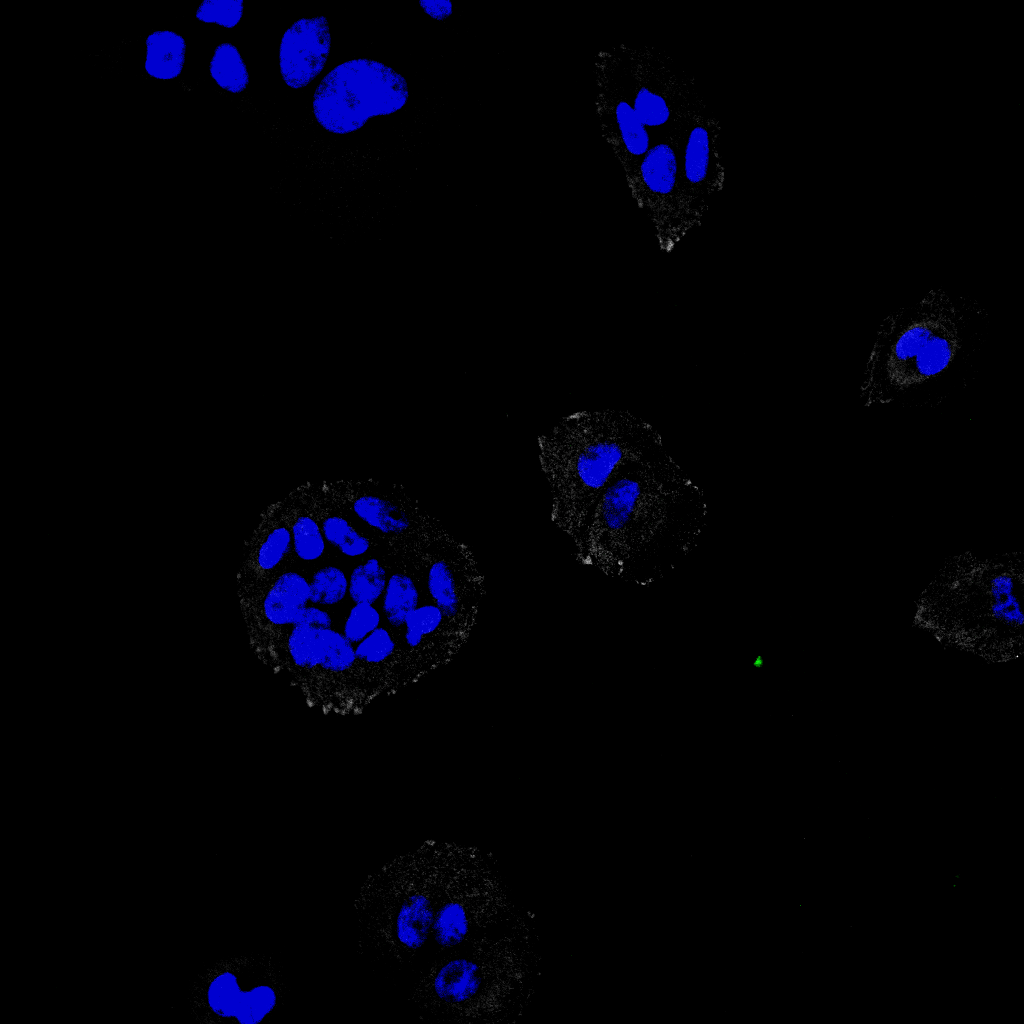

Supplement: Source data 1. — These files are the best ranked (ranked 0) predictions generated by AlphaFold of HEV ORF1, its associated point mutants, and the hepatitis A virus (HAV) 3 C protease. [file elife-80529-data1.zip › Figure 7 Source Data/Merge_Mock_duplicate1_40x_C_20220405.nd2 (RGB).tif]

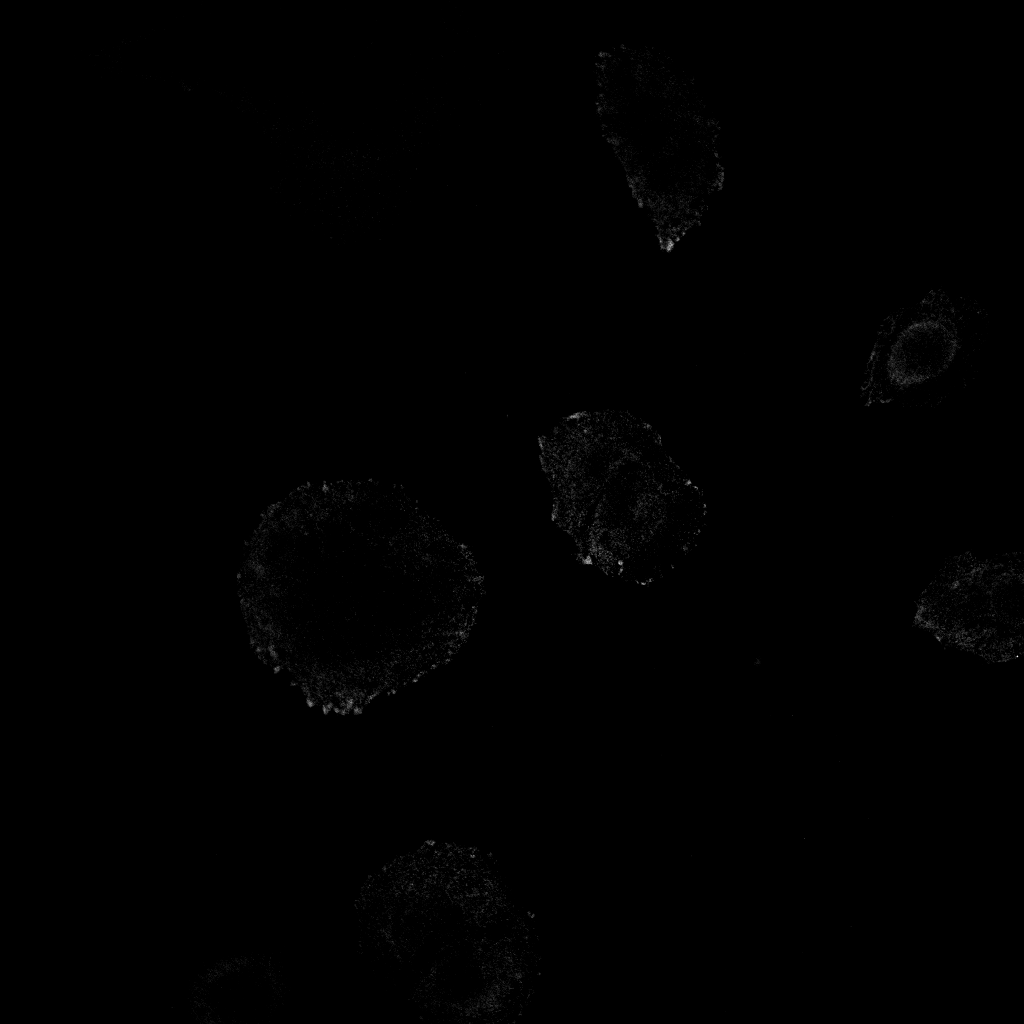

Supplement: Source data 1. — These files are the best ranked (ranked 0) predictions generated by AlphaFold of HEV ORF1, its associated point mutants, and the hepatitis A virus (HAV) 3 C protease. [file elife-80529-data1.zip › Figure 7 Source Data/HA_C3-Mock_duplicate1_40x_C_20220405.tif]

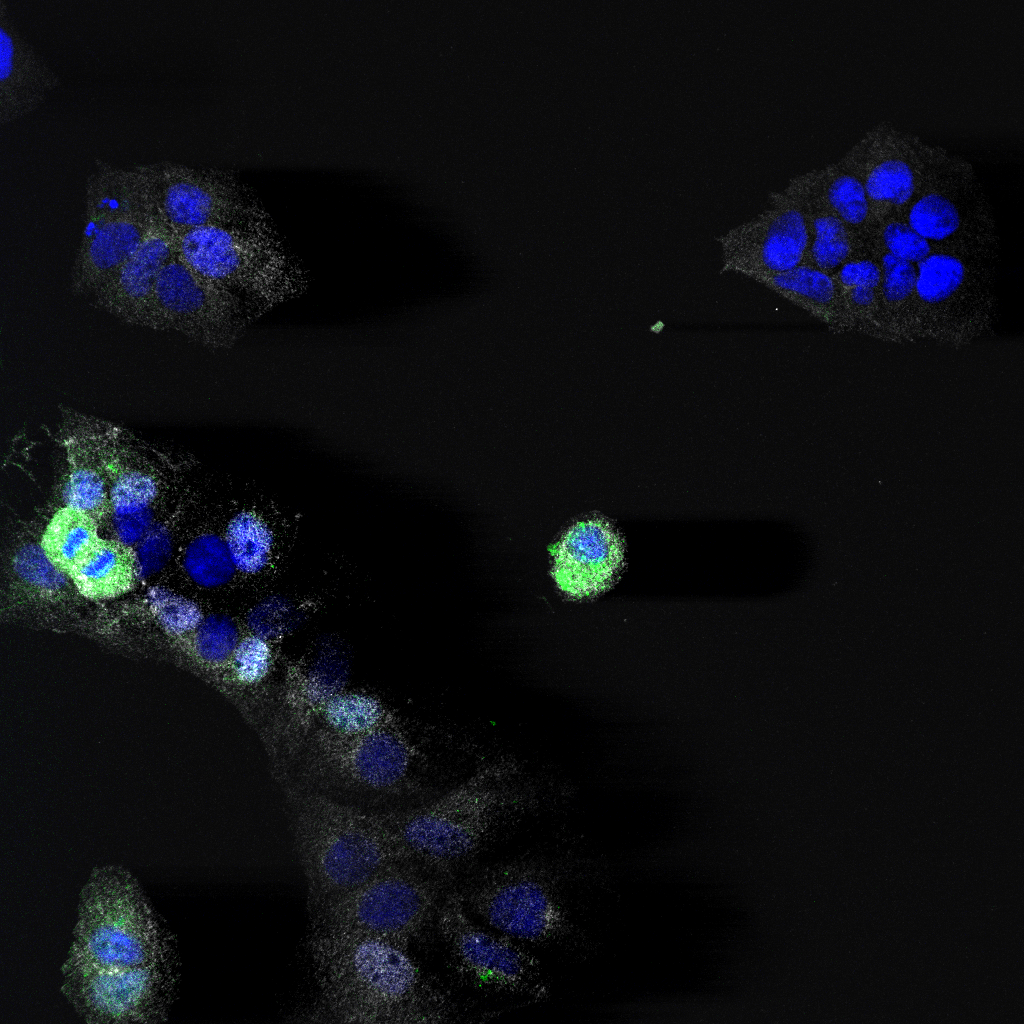

Supplement: Source data 1. — These files are the best ranked (ranked 0) predictions generated by AlphaFold of HEV ORF1, its associated point mutants, and the hepatitis A virus (HAV) 3 C protease. [file elife-80529-data1.zip › Figure 7 Source Data/Merge_L-HA-L_D248A_A_20220405.nd2 (RGB).tif]

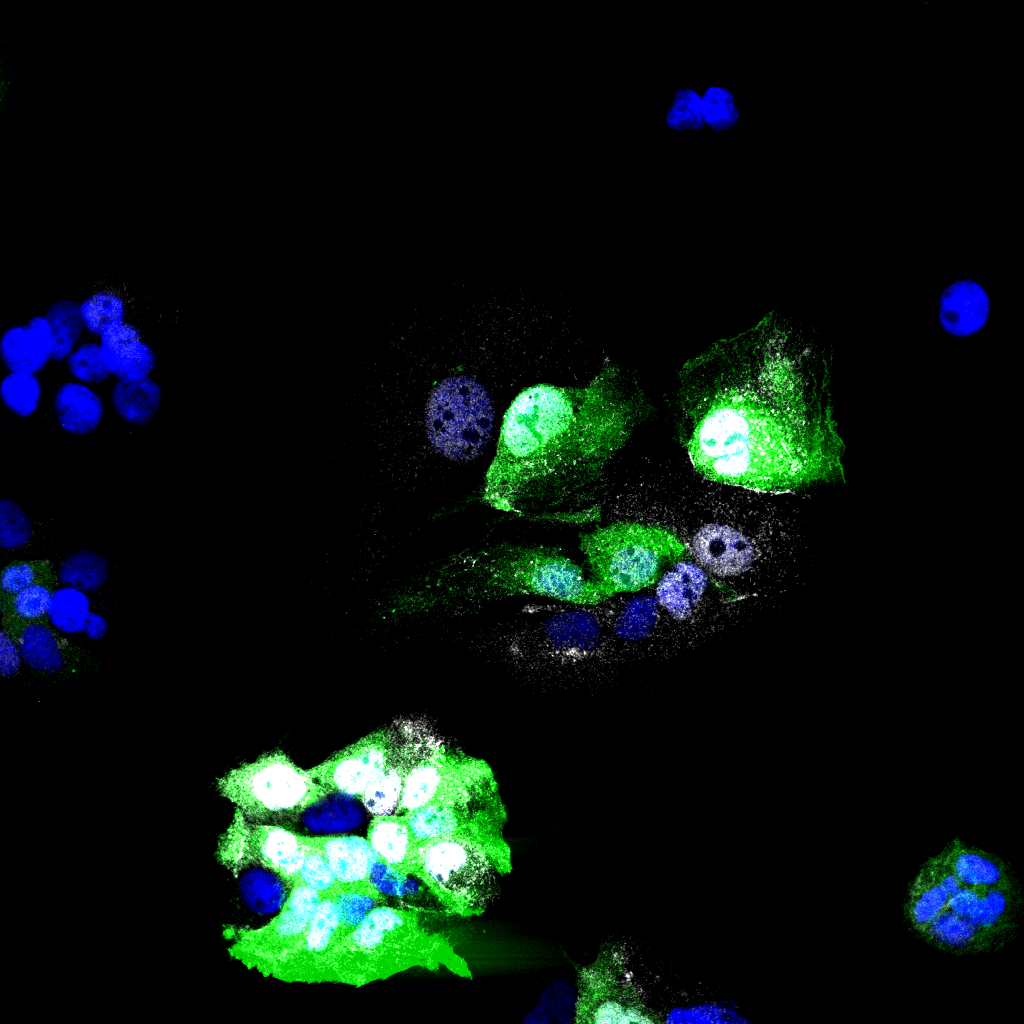

Supplement: Source data 1. — These files are the best ranked (ranked 0) predictions generated by AlphaFold of HEV ORF1, its associated point mutants, and the hepatitis A virus (HAV) 3 C protease. [file elife-80529-data1.zip › Figure 7 Source Data/Merge_L-Ha-L_C563A_C_20220406.nd2 (RGB).tif]

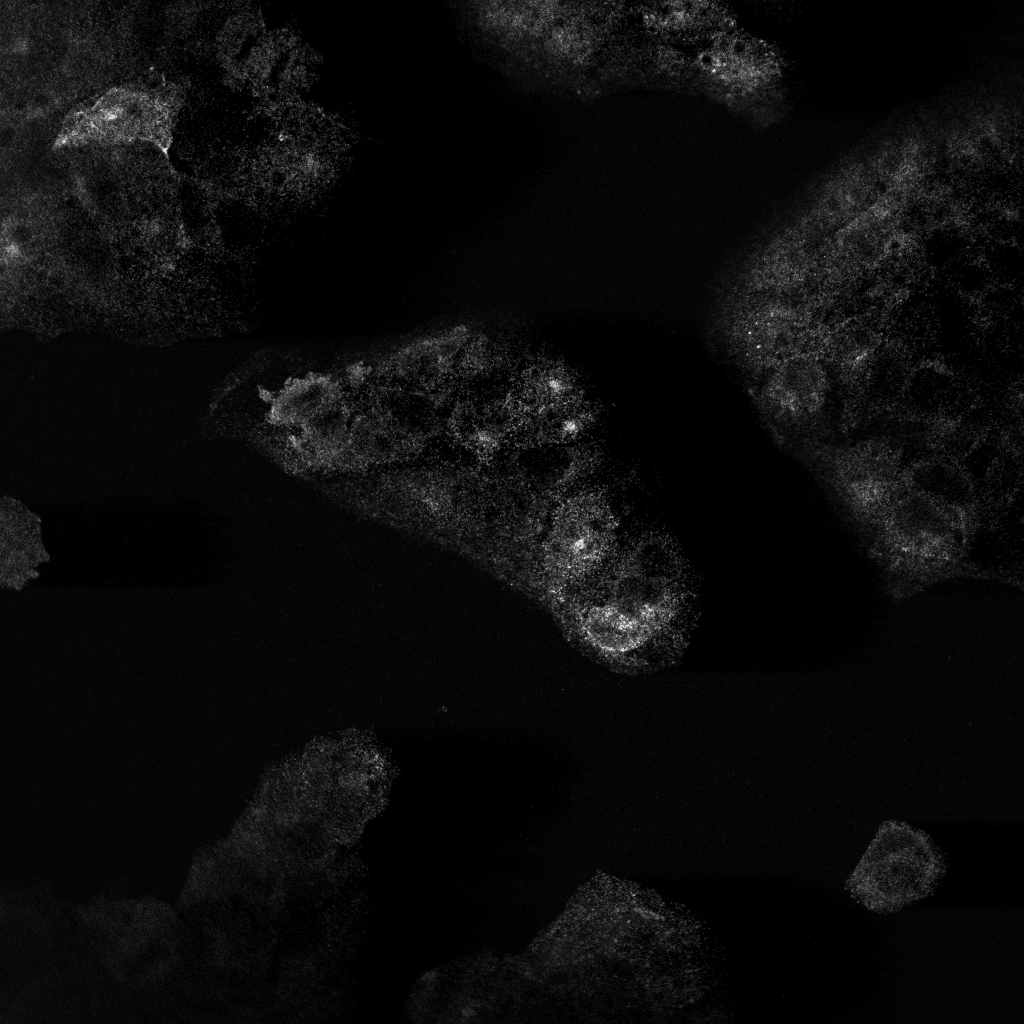

Supplement: Source data 1. — These files are the best ranked (ranked 0) predictions generated by AlphaFold of HEV ORF1, its associated point mutants, and the hepatitis A virus (HAV) 3 C protease. [file elife-80529-data1.zip › Figure 7 Source Data/HA_C3-L-HA-L_C483A_40x_F_20220405.tif]

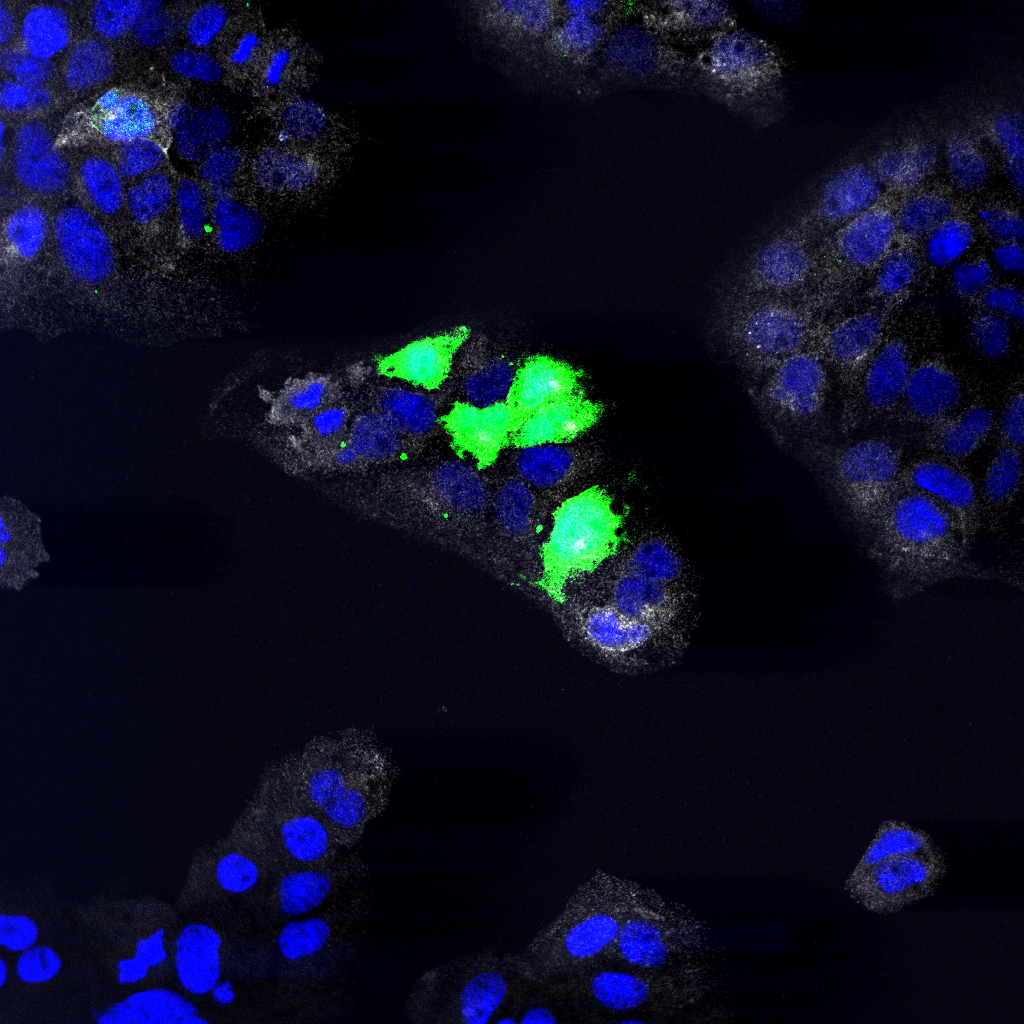

Supplement: Source data 1. — These files are the best ranked (ranked 0) predictions generated by AlphaFold of HEV ORF1, its associated point mutants, and the hepatitis A virus (HAV) 3 C protease. [file elife-80529-data1.zip › Figure 7 Source Data/Merge_L-HA-L_C483A_40x_F_20220405.nd2 (RGB).tif]
